# Changes in Acute ED Visits by Race/Ethnicity During the Early COVID-19 Pandemic

**DOI:** 10.1007/s10903-023-01499-w

**Published:** 2023-06-03

**Authors:** Celina Morales, Tim A. Bruckner, Senxi Du, Andrew Young, Annie Ro

**Affiliations:** 1 Department of Population Health and Disease Prevention, University of California Irvine, 653 E. Peltason Dr, Irvine, CA 92697-3957 USA; 2 Department of Health, Society, and Behavior, University of California Irvine, Irvine, CA USA; 3 Department of Medicine, University of Southern California, Los Angeles, CA USA; 4 Harbor UCLA Medical Center, Los Angeles, CA USA

**Keywords:** Acute medical emergencies, Ethnic minorities, Coronavirus pandemic, Safety net hospital

## Abstract

Emergency department (ED) visits for conditions unrelated to the Coronavirus Disease 2019 (COVID-19) pandemic decreased during the early pandemic, raising concerns about critically ill patients forgoing care and increasing their risk of adverse outcomes. It is unclear if Hispanic and Black adults, who have a high prevalence of chronic conditions, sought medical assistance for acute emergencies during this time. This study used 2018–2020 ED visit data from the largest safety net hospital in Los Angeles County to estimate ED visit differences for cardiac emergencies, diabetic complications, and strokes, during the first societal lockdown among Black and Hispanic patients using time series analyses. Emergency department visits were lower than the expected levels during the first societal lockdown. However, after the lockdown ended, Black patients experienced a rebound in ED visits while visits for Hispanics remained depressed. Future research could identify barriers Hispanics experienced that contributed to prolonged ED avoidance.

## Introduction

During the earliest days of the Coronavirus Disease 2019 (COVID-19) pandemic, United States (US) healthcare centers saw a dramatic reduction in emergency department (ED) visits for non-COVID-19 care [1–7]. Several hospitals reported up to a 50–60% decrease in ED visits during the early pandemic period [5, 7, 8], including visits which typically require urgent inpatient procedures. Further, during March, April, and May of 2020 there was a 23% reduction in visits for heart attacks, a 20% reduction for strokes, and a 10% reduction for hyperglycemic crises nationwide [9].

As many acute medical emergencies are the result of untreated chronic disease, it is unlikely the pandemic reduced chronic disease prevalence but rather that it increased medical avoidance for serious conditions unrelated to COVID-19 [10–13]. The decrease in ED utilization for acute conditions among non-COVID-19 patients [7, 9, 14–16] raises many concerns about critically ill patients forgoing care and increasing their risk of adverse outcomes and even death [17]. Cardiac emergencies [18], strokes [19], and diabetic emergencies [20] need time sensitive medical interventions to prevent further morbidity and mortality. Cardiac arrests and cardiac arrest deaths unrelated to COVID-19 increased in non-clinical settings [21, 22] which suggests that people were not seeking care in medical facilities but instead waited until their conditions became severe before calling for medical help.

While the decline in acute ED visits has been well-documented, there are still questions about which populations experienced significant declines, and by extension, may be at risk for subsequent adverse outcomes. Some empirical evidence suggests that the overall decline in ED utilization was concentrated among certain race/ethnic groups who already face high medical needs. In the early days of the pandemic, there was a large reduction in non-COVID-19 admissions for ZIP codes which were majority-Hispanic, majority-Black, or high poverty [2, 6]. However, the in-hospital mortality for non-COVID-19 conditions was high among patients from these subgroups [2], suggesting that these patients might have avoided seeking healthcare services until the condition substantially worsened. Existing literature has not yet examined whether ED visits for non-COVID acute conditions, which have more negative health ramifications, followed similar racial/ethnic and income patterns as did overall ED use. Past studies examining ED visits during the COVID-19 pandemic often use a “stacked calendar” approach which does not consider seasonal variations in ED visits [23].

It is additionally unclear how long this downturn in ED utilization persisted. Understanding the duration of reduced ED care has important health implications, as potential health effects due to the drop in ED visits for acute conditions could be mitigated if visits rebounded after the initial downturn [2, 9, 14–16, 24, 25], suggesting that people were actually delaying instead of forgoing needed care. The empirical evidence in this area does not converge on a specific date of ED “return”, with some reporting returns as early as late-April [15] and others documenting a return in mid-summer 2020 [2, 25]. Few studies, however, examine whether this rebound was experienced equally across all racial/ethnic groups. Considering that non-COVID-19 medical admissions among Black, Hispanic, and high poverty populations increased but were still below pre-pandemic counts in the later pandemic periods [2], ED visits for acute conditions among historically marginalized populations likely followed a similar trend. We examine how ED visits for cardiac emergencies, stroke, and diabetes complications at a large, urban safety net hospital in Los Angeles County during the COVID-19 pandemic changed among Hispanic and Black patients during the first stay-at-home orders and whether the trend continued as stay-at-home orders were lifted using time series analyses.

## Methods

### Data Source

We used electronic health records (EHR) extracted for ED visits from January 5, 2018, to December 31, 2020, from the Los Angeles County + USC Medical center (LAC + USC), a 600-bed safety-net hospital and the largest safety-net hospital of Los Angeles County. The EHR data were extracted from the LAC + USC Medical Center’s Cerner PowerInsight clinical reporting tool. The patient diagnoses codes were inputted for administrative purposes, including patient care and billing, and were assigned by trained coders at the medical center based on physician documentation. All project activities were reviewed and approved by the University of Southern California Institutional Review Board (HS-19-00890), which served as a reliance for the University of California, Irvine Institutional Review Board.

### Measures

Cardiac emergencies, diabetic complications, and strokes were identified using the Clinical Classifications Software (CCS) for the International Classification of Diseases, Tenth Revision (ICD-10) diagnosis codes. Cardiac emergencies were defined as acute myocardial infarctions (CCS code 100), coronary atherosclerosis and other heart disease (CCS code 101), cardiac dysrhythmias (CCS code 106), and cardiac arrest and ventricular fibrillation (CCS code 107). Patients with diabetic complications were defined as having diabetes mellitus with complications (CCS code 50) including ketoacidosis, hypoglycemia, hyperglycemia, skin ulcers, macular edema, or chronic kidney disease. Stroke emergencies were defined as acute cerebrovascular disease (CCS code 109), occlusion or stenosis of precerebral arteries (CCS code 110) and transient cerebral ischemia (CCS code 112). These emergency conditions were selected because they require timely healthcare to prevent additional health complications and death. These three conditions have also been previously examined using national syndromic surveillance program data to determine the level of ED utilization during the pandemic for specific acute conditions [9]. Some conditions, such as cardiac arrest, are often fatal if not immediately treated. Alternatively, for conditions which are serious but not immediately life threatening, the symptoms may be temporarily managed without medical assistance, but often lead to significant morbidities and even mortality without proper medical intervention. We combined all three acute medical conditions of interest into a single composite variable (cardiac emergencies, diabetic complications, and stroke conditions) for the time series analyses, as counts for the individual conditions were low.

### Statistical Methods

Our analyses included the 156 weeks between January 5, 2018, and December 31, 2020. We designated the early pandemic period as the eight weeks between March 13, 2020, and May 7, 2020, which started when the Trump Administration issued the national emergency order and ended when California started relaxing social distancing restrictions and businesses were reopening [26]. We designated the later (i.e., “rebound”) pandemic period to be the subsequent eight-week period from May 8, 2020, to July 2, 2020. We started with descriptive analyses comparing average weekly visits and percent changes for ED visits during our early and later pandemic periods to the same weeks in 2019 for cardiac emergencies, diabetic complications, and stroke conditions separately and for the composite variable.

To test whether the early and later pandemic periods represented anomalous ED utilization trends than would be expected, we conducted an interrupted time series analysis using autoregressive integrated moving average (ARIMA) [27]. Our main independent variable was a binary variable to indicate weeks in our periods of interest versus all other weeks. The ARIMA methods, developed by Box and Jenkins [27], identify and remove any seasonal trends in ED visits, or autocorrelation, by separately estimating three parts in the model: the autoregressive (AR), the differencing (I), and the moving average components (MA). These components account for well-documented temporal patterning (e.g., trend, seasonality) in 2018–2020 ED visits in the weeks leading up to our period of interest. We ran all models twice: once for the early pandemic period and again for the later period. All analyses were disaggregated by Black and Hispanic race/ethnicity. The descriptive analyses were conducted in Stata Statistical Software (version 17.0, StataCorp., College Station, TX) and ARIMA analyses were conducted using SCA Statistical System Software (version 5.4.6, SCA Corp., Villa Park, IL).

## Results

**Fig. 1:**
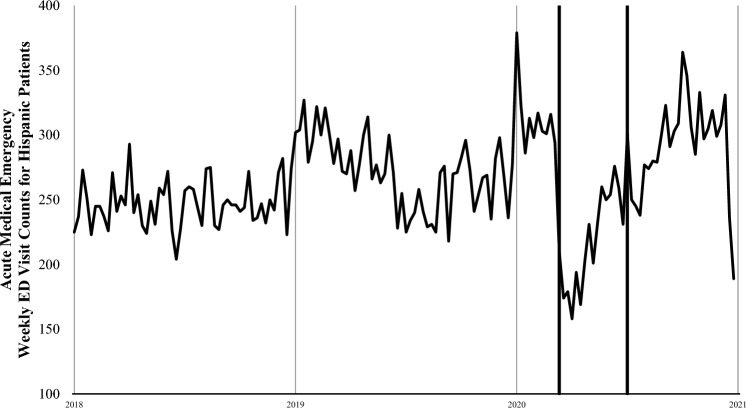
Weekly ED visit counts for acute medical emergencies among Hispanic patients at LAC+USC Medical Center during January 5, 2018, to December 31, 2020. The thick lines indicate the period between March 13 and July 2, 2020. The acute medical emergencies include cardiac emergencies, diabetic complications, and strokes

**Fig. 2:**
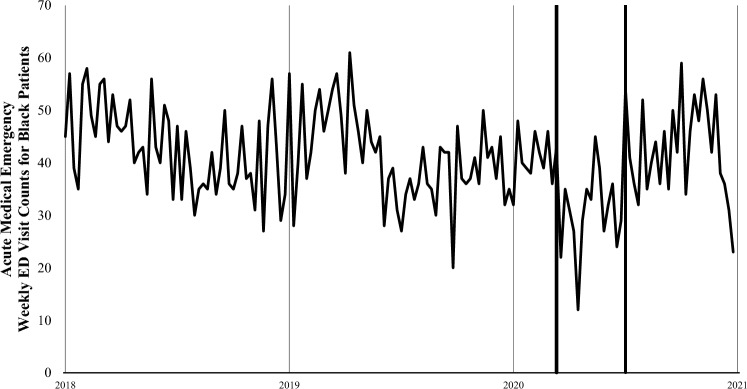
Weekly ED visit counts for acute medical emergencies among Black patients at LAC+USC Medical Center during January 5, 2018, to December 31, 2020. The thick lines indicate the period between March 13 and July 2, 2020. The acute medical emergencies include cardiac emergencies, diabetic complications, and strokes

For the three medical conditions of interest there were a total of 4864 ED visits between March 13 and July 2, 2020: 2,596 were for cardiac emergencies (53.4%), 2,014 were for diabetic complications (41.4%), and 254 were for strokes (5.2%). During the early pandemic period, total ED visits were 48% lower than in the same period in 2019 (2020, 14,788 visits vs 2019, 28,689 visits) and a larger proportion of males visited the ED (2020, 59% vs 2019, 53%) (Table 1). This difference narrowed slightly during the later pandemic period but was still 35% lower compared with 2019 (2020, 17,849 visits vs 2019, 27,524 visits). We plotted ED visit counts for acute medical emergencies among Hispanic patients (Fig. 1) and Black patients (Fig. 2), for every 7-day period between January 5, 2018, and December 31, 2020. Figures 1 and 2 show an abrupt decline in ED visits for acute medical emergencies among Hispanic and Black patients beginning on March 13, 2020. Subsequent weekly ED visit counts were lower than in the same weeks of 2018 and 2019.

**Table 1:** LAC + USC Medical Center patient demographics across the first four months of the COVID-19 pandemic in the US and the comparison period in 2019

	Early pandemic period March 13, 2020–May 7, 2020 (N = 14,788)	Early pandemic comparison period March 15, 2019–May 9, 2019 (N = 28,689)	Later pandemic period May 8, 2020–July 2, 2020 (N = 17,849)	Later pandemic comparison period May 10, 2019–July 4, 2019 (N = 27,524)
Patient demographics				
Race/Ethnicity				
Non-Hispanic White	560 (3.8%)	923 (3.2%)	700 (3.9%)	865 (3.1%)
Non-Hispanic Asian	489 (3.3%)	998 (3.5%)	645 (3.6%)	999 (3.6%)
Non-Hispanic Black	1,940 (13.1%)	3,004 (10.5%)	2,063 (11.6%)	2,931 (10.6%)
Non-Hispanic Other	1,630 (11.0%)	3,243 (11.3%)	1,817 (10.2%)	3,147 (11.4%)
Hispanic	10,169 (68.8%)	20,521 (71.5%)	12,624 (70.7%)	19, 582 (71.1%)
Gender				
Female	6,058 (41.0%)	13,591 (47.4%)	7,651 (42.9%)	12,862 (46.7%)
Male	8,730 (59.0%)	15,098 (52.6%)	10,198 (57.1%)	14,662 (53.3%)
Age (Mean ± SD)	43.6 ± 17.5	41.7 ± 19.1	44.0 ± 17.2	42.5 ± 18.8
Total acute emergencies	2,144 (14.5%)	3,354 (11.7%)	2,720 (15.2%)	3,012 (10.9%)
Cardiac emergency	1,177 (7.9%)	1,854 (6.5%)	1,419 (7.9%)	1,590 (5.8%)
Diabetic complications	868 (6.1%)	1,315 (4.6%)	1,146 (6.4%)	1,276 (4.6%)
Stroke	99 (0.6%)	185 (0.6%)	155 (0.9%)	146 (0.5%)

### Early Pandemic Period (March 13, 2020, to May 7, 2020)

In comparison to 2019, there was a 33% reduction in total acute emergencies for Hispanics and a 36% reduction for Blacks (Table 2). Among Hispanics specifically, there was a steep reduction in ED visits for cardiac emergencies (− 35%), diabetic complications (− 30%), and strokes (− 46%). Similarly, among Blacks there was a 39% reduction in ED visits for cardiac emergencies, 41% reduction in ED visits for diabetic complications, and a 65% reduction in ED visits for strokes.

**Table 2:** Comparison of percentage change in ED visits for acute medical emergencies between 2019 and 2020 by race/ethnicity

	Early pandemic period March 13, 2020–May 7, 2020		Early pandemic comparison period March 15, 2019–May 9, 2019		
	n	Average weekly visits	n	Average weekly visits	% Change
Hispanic					
Cardiac emergency	751	93.87	1,152	144.00	− 35
Diabetic complications	699	87.37	998	124.75	− 30
Stroke	67	8.37	125	15.62	− 46
Black					
Cardiac emergency	149	18.62	244	30.50	− 39
Diabetic complications	79	9.87	135	16.87	− 41
Stroke	6	0.75	17	2.12	− 65

	Later pandemic period May 8, 2020–July 2, 2020		Later pandemic comparison period May 10, 2019–July 4, 2019		
	n	Average weekly visits	n	Average weekly visits	% Change
Hispanic					
Cardiac emergency	954	119.25	1,024	198.75	− 7
Diabetic complications	930	116.25	1,019	127.37	− 9
Stroke	79	9.87	87	10.87	− 9
Black					
Cardiac emergency	152	19.00	199	24.87	− 24
Diabetic complications	91	11.37	100	12.50	− 9
Stroke	22	2.75	17	2.12	29

The time-series analyses results for Hispanic and Black patient ED visits during the early and later pandemic periods are shown in Table 3. Acute condition ED visits for both Hispanic and Black patients fell below expected levels during the early pandemic period of the COVID-19 pandemic shutdown. There was a 23% percent reduction in ED visits for the three acute conditions among Hispanic patients [coefficient = − 61.09; Standard error (Std. Error) = 16.8); p-value < 0.001] and 26% reduction among Black patients (coefficient = − 10.71; Std. Error = 3.30; p-value < 0.01). These coefficients translate to 489 fewer ED visits for Hispanics and 86 fewer visits for Blacks during the time period.

**Table 3:** Autoregressive integrated moving average (ARIMA) time series analyses results predicting the count of weekly emergency department visits among Hispanic and Black patients, separately, at LAC + USC Medical Center during the early and later COVID-19 pandemic lockdown periods, January 5, 2018, to December 31, 2020

Parameter	Hispanic patients					
	Early pandemic period March 13, 2020–May 7, 2020			Later pandemic period May 8, 2020–July 2, 2020		
	Coefficient	Standard Error	p-value	Coefficient	Standard Error	p-value
Period of interest	− 61.10	16.802	< .001	− 38.76	18.403	< .05
AR (1)	0.46	0.081	< .001	0.70	0.059	< .001
AR (3)	0.23	0.086	< .01			
AR (5)	0.20	0.087	< .05			
Percent change	22.8%			14.6%		

	Black patients					
	Early pandemic period March 13, 2020–May 7, 2020			Later pandemic period May 8, 2020–July 2, 2020		
	Coefficient	Standard error	p-value	Coefficient	Standard error	p-value
Period of interest	− 10.72	3.309	< .01	− 1.67	3.742	.653
AR (3)	0.28	0.075	< .001	0.27	0.075	< .001
AR (4)	0.30	0.075	< .001	0.31	0.076	< .001
MA (2)				− 0.19	0.084	< .05
Percent change	26.1%			4.1%		

### Later Pandemic Period (May 8, 2020, to July 2, 2020)

Visits for acute conditions appeared to slowly recover in the later pandemic period but did not reach pre-pandemic levels (Table 2). Emergency department visits for cardiac emergencies (− 7%), diabetic complications (− 9%), and strokes (− 9%) almost closed the gap between 2020 and 2019 among Hispanics. Alternatively, there was a full rebound for stroke visits among Black patients, and there were 29% more stroke visits than in the same period in 2019. Yet, a 9% reduction in visits for diabetic complications and 24% reduction in visits for cardiac emergencies remained for Black patients.

In our ARIMA results for the later lockdown period, ED visits for the total medical conditions were reduced by 14.6% for Hispanic patients (coefficient = − 38.75; Std. Error = 18.40; p-value < 0.05) and 4.1% for Black patients (coefficient = − 1.66; Std. Error = 3.74; p-value = 0.653), while accounting for autocorrelation. The coefficient for Black patients was not significantly different from zero, meaning that the average number of ED visits for acute conditions during the post lock-down was not different from previous weeks.

## Discussion

Our study examined the reduction in ED visits at a Los Angeles safety-net hospital during the early COVID-19 pandemic for Black and Hispanic patients. Consistent with other work [9, 14, 15], we discovered a sharp decline in ED visits in Spring 2020 for cardiac emergencies, strokes, and diabetic complications. Our study also reveals important racial/ethnic trends in ED utilization, notably that Black patients experienced a rapid rebound in ED visits for acute conditions between May and July 2020. By contrast, Hispanic patients showed lower-than-expected ED use even after the stay-at-home orders were lifted. These differences in health behaviors suggest Black patients were benefiting from undocumented protective factors, whereas Hispanic patients were continuing to delay visiting EDs for medical help. These race-specific ED responses after the initial set of societal restrictions ended warrants further scholarly attention.

We do not interpret this acute drop in ED visits in Spring 2020 as a reduction in disease incidence because these medical emergencies often result from uncontrolled chronic conditions [28]. The reduction may be better explained by fear [22], worry [12], and perceived level of threat [29] of COVID-19 infection from healthcare settings, which may also suggest distrust in medical practices. Hispanic and Black communities were disproportionately affected by COVID-19 during the first year of the pandemic [30, 31] and patients might have avoided EDs to prevent COVID-19 infection [10]. While avoidance of urgent medical care was widespread [11, 12, 21], it was more common among Black [11, 12] and Hispanic [11] adults and among people with chronic health conditions [11, 12]. Between late-May and early-June 2020, a survey by the American Heart Association [10] found over half of all Hispanic adults and nearly half of all Black adults, would be too fearful of going to the hospital if they were experiencing a stroke or heart attack, because they feared being infected with COVID-19. Our results provide concrete evidence that Black and Hispanic patients avoided the ED for these serious medical conditions.

This ED avoidance is especially problematic for Black and Hispanic patients, as they have a high prevalence of the underlying conditions [32–34] that trigger many of these acute visits. It is unknown if the reduction in ED visits for cardiac emergencies, diabetic complications, and strokes in this study among Black and Hispanic patients is associated with an increase in deaths. We suggest that future research consider whether mortality related to these conditions increased as well.

Black patients experienced a rapid rebound in ED visits for acute conditions between May and July 2020, but Hispanic patients continued to see lower-than-expected ED use after the stay-at-home orders were lifted. In California, lockdown restrictions were lifted in early May 2020 [26], which may have eased fears in patients who were previously avoiding medical care. A similar study examining acute myocardial infarctions in Southern California also reported a rebound in visits when California reopened in early May [25]. Differences in perceived susceptibility of COVID-19 infection among racial/ethnic groups could explain the difference in rebound among Hispanic and Black patients. For instance, although both Hispanics and Blacks reported fear of COVID-19 in late-May and early-June 2020, a larger proportion of Hispanics were fearful of being infected with COVID-19 in a hospital setting [10].

Another possible explanation for the difference in ED rebound magnitude between Black and Hispanic patients is help seeking behaviors, namely the use of telemedicine. The empirical evidence is mixed but some studies found Black patients utilized telemedicine more than non-Black patients during the early pandemic [35, 36], which suggests that Black patients were avoiding in-person visits but continuing to monitor their health remotely. In contrast, Hispanic patients were less likely to utilize telemedicine during the early pandemic [35] and in the later pandemic [37] compared to non-Hispanic patients, which suggests Hispanic patients were not receiving any medical advice during the lockdown period and the following months. Our study did not examine telemedicine insurance coverage, access, and utilization by race/ethnicity, but a shift to telemedicine for medical assistance among Black patients during the lockdown (March–May 2020) could explain the decrease in in-person ED utilization during the early pandemic when lockdowns were enforced, and then the strong rebound in in-person ED visits when the lockdown was lifted in May 2020.

Our paper addresses important questions about the health implications of the widely studied decline in ED use immediately following the COVID-19 national emergency declaration in March 2020 by better characterizing the nature of this slowdown by race/ethnicity and the stability of this trend after stay-at-home orders were lifted. These findings can help researchers and practitioners understand potential health impacts of the well-documented drop in ED visits by identifying most affected patient groups, further characterizing gaps in healthcare utilization, and the magnitude of the rebound in ED use to compensate for the downturn. Importantly, we also improve upon earlier work by using time-series methods to control for well-characterized pre-COVID-19 patterns in ED utilization.

### Limitations

There are a few limitations to our study. As we do not have data outside of the ED setting, we do not know whether patients who avoided the ED sought help elsewhere, or if they died with EMS or at home. Moreover, our study did not examine mortality data and therefore it is unclear if there was an increase in deaths due to our medical conditions of interest. Although lack of mortality data was a limitation, documenting the delay in care for these conditions is a strength of the study since delayed treatment can lead to additional complications which adversely influence patients’ lives and burden the healthcare system. These study findings may not generalize to all regions because the population analyzed were patients visiting an ED at a safety net hospital in an urban location. However, these results may be applicable to other safety net hospitals located in regions with a high social vulnerability index. Our study also used a composite variable for the timeseries analysis due to the low individual counts, yet despite this limitation the time-series analysis was an important strength of the study, and we were able to adjust for seasonal trends in ED visits using multi-year data. Further, this study fills the significant gap in research by investigating ED utilization for urgent medical conditions among historically marginalized and low-income groups.

## Conclusions

Among Hispanic and Black patient, ED visits for cardiac emergencies, diabetic complications, and strokes, declined significantly during the early COVID-19 pandemic period. After societal restrictions were loosened in May 2020, however, there was a gradual rebound among Black patients. The magnitude of the rebound was less pronounced in Hispanic patients. Further exploration into health behaviors is needed to determine what factors motivated Hispanics to continue to avoid medical assistance for time-sensitive, life-threatening conditions, as well as any protective factors that motivated Black patients to seek care after the initial decline. To date, new information is being discovered about the COVID-19 disease and much is still unknown. It is possible that highly contagious COVID-19 subvariants can cause a surge in cases and deaths, which overwhelm EDs and compel governments to reinforce lockdowns similar to March 2020. Health agencies should communicate the importance of seeking medical assistance at EDs for cardiac emergencies, diabetic complications, and strokes, even during lockdowns to minimize treatable conditions and avoidable deaths. Addressing these disparities and encouraging patients to seek medical care for urgent conditions can mitigate the burden on the healthcare system.
